# Key characteristics of carcinogens meet hallmarks for prevention-cutting the Gordian knot

**DOI:** 10.3389/fonc.2024.1420687

**Published:** 2024-09-10

**Authors:** Sasi S. Senga, William H. Bisson, Annamaria Colacci

**Affiliations:** ^1^ Nuffield Department of Medicine, University of Oxford, Oxford, United Kingdom; ^2^ Integrative Toxicology and Cancer Prevention, Durham, NC, United States; ^3^ Agency for Prevention, Environment and Energy, Emilia-Romagna (Arpae), Bologna, Italy; ^4^ Alma Mater Institute on Healthy Planet – University of Bologna, Bologna, Italy

**Keywords:** KCCs, cancer hallmarks, chemical carcinogens, cancer process, regulatory toxicology, precise toxicology, environmental exposure, key carcinogens characteristics

## Abstract

The complexity of cancer requires a comprehensive approach to understand its diverse manifestations and underlying mechanisms. Initially outlined by Hanahan and Weinberg in 2000 and updated in 2010, the hallmarks of cancer provide a conceptual basis for understanding inherent variability in cancer biology. Recent expansions have further elucidated additional hallmarks, including phenotypic plasticity and senescent cells. The International Agency for Research on Cancer (IARC) has identified the key characteristics of carcinogens (KCCs) to evaluate their carcinogenic potential. We analyzed chemicals of concern for environmental exposure that interact with specific receptors to induce genomic instability, epigenetic alterations, immune suppression, and receptor-mediated effects, thereby contributing to chronic inflammation. Despite their varying degrees of carcinogenicity, these chemicals have similar KCC profiles. Our analysis highlights the pivotal role of receptor binding in activating most other KCCs, underscoring their significance in cancer initiation. Although KCCs are associated with early molecular or cellular events, they do not encompass processes directly linked to full cellular malignancy. Thus, there is a need to integrate clear endpoints that anchor KCCs to the acquisition of a complete malignant phenotype into chemical testing. From the perspective of toxicology and cancer research, an all-encompassing strategy that incorporates both existing and novel KCCs and cancer hallmarks is essential to enable the targeted identification of prevalent carcinogens and facilitate zone-specific prevention strategies. To achieve this goal, collaboration between the KCC and cancer hallmarks communities becomes essential.

## 1 Introduction

Cancer cells interact with a complex microenvironment, underscoring the inherent variability in cancer. Hanahan and Weinberg captured this complexity by outlining the hallmarks of cancer in 2000 and updating them in 2010 (1, 2). Senga and Grose expanded the hallmarks of cancer in 2021 by introducing additional hallmarks, such as dedifferentiation/transdifferentiation, epigenetic dysregulation, altered microbiome, and altered neuronal signaling (3). In 2022, Hanahan proposed the unlocking of phenotypic plasticity, non-mutational epigenetic reprogramming, senescent cells, and polymorphic microbiomes as additional hallmarks and emerging characteristics (4)

The *exposome concept* was introduced as a new paradigm for understanding and measuring all non-genetic factors that influence individuals throughout their lives, serving as a counterpart to the genome (5) This concept underscores the importance of capturing diverse environmental exposures, including chemical, biological, and psychosocial factors, to comprehensively assess their collective impact on health outcomes, such as carcinogenesis (5, 6). The Halifax Project Task Force, a pioneering effort in 2013, employed the original eleven hallmarks to evaluate the carcinogenic potential of environmental chemical mixtures and low-dose exposures (7). This initiative highlighted the critical need for robust data on environmental toxin exposures to elucidate their role in cancer development. Concurrently, an expert panel workshop convened by the International Agency for Research on Cancer (IARC) identified for over hundred Group 1 cancer hazards ten key characteristics of carcinogens (KCCs), offering a structured and comprehensive approach to identify, assess and classify the carcinogenic potential of various environmental agents (8, 9) (**Table 1**).

**Table 1:** Key characteristics of carcinogens and evolution of cancer hallmarks from 2000 to 2022.

Key Carcinogens Characteristics (Smith et al, 2016)
1. electrophilic or can be metabolically activated 2. Is genotoxic 3. Alters DNA repair or causes genomic instability 4. Induces epigenetic alterations 5. Induce oxidative stress 6. Induces chronic inflammation 7. Is immunosuppressive 8. Modulates receptor-mediated effects 9. Causes immortalization 10. Alters cell proliferation, cell death or nutrient supply
Cancer Hallmarks (Hanahan and Weinberg, 2000)
− Self-Sufficiency in Growth Signals − Insensitivity to Antigrowth Signals − Evading Apoptosis − Limitless Replicative Potential − Sustained Angiogenesis − Tissue Invasion and Metastasis − Genome Instability
Cancer Hallmarks: the next generation (Hanahan and Weinberg, 2011)
− Genomic instability and evolution − Tumor-Promoting Inflammation − Reprogramming Energy Metabolism − Evading Immune Destruction
Cancer Hallmarks: the new testament (Senga and Grose, 2021)
− Dedifferentiation/Transdifferentiation − Epigenetic dysregulation − Altered microbiome − Altered neuronal signaling
Cancer Hallmarks: new dimensions (Hanahan, 2022)
− Unlocking Phenotypic Plasticity − Nonmutational Epigenetic Reprogramming − Polymorphic microbiomes − Senescent cells

The IARC Preamble, as amended in 2019 (10), and the upcoming updated Handbook of the Report on Carcinogens (RoC) by the US National Toxicology Program (NTP (11) to develop monographs underscores the importance of refining current methods and/or adding novel methods to mechanistically identify and assess cancer hazards. This emphasizes the need of utilizing an integrated approach that prioritizes causation over association and incorporates both KCCs and cancer hallmarks for precision environmental health.

To enhance the understanding of the relationship between KCCs and cancer hallmarks, we analyzed a group of chemicals of environmental concern These were classified into different ranks of carcinogenicity by the IARC (12) and NTP (13) including polycyclic aromatic hydrocarbons (PAHs), perfluoroalkyl substances (PFASs), phthalates, and endocrine disruptors (EDC), such as organophosphate (OPFRs) and halogenated (HRF) flame retardants, with arsenic as a paradigmatic representative of heavy metals (**Supplementary Table S1**).

PAHs and their nitro-derivatives (NPAHs) are among the most significant air pollutants and are implicated in respiratory pathologies, including cancer. PFASs are associated with a spectrum of health concerns, including potential adverse effects on immune function and metabolism. Their carcinogenic properties have recently been revaluated based on new data and KCCs (14).

Phthalates have emerged as significant environmental contaminants, potentially linked to the escalation of various health issues, including reproductive disorders. OPFRs and HRFs are persistent pollutants used to reduce the flammability of various materials including plastics, textiles, and foam products. Some OPFRs such as tris(2,3-dibromopropyl) phosphate (TDBPP) are considered potential human carcinogens. Arsenic is recognized as a significant environmental hazard and is implicated in a range of health issues including cancer. These substances interact with significant receptors, whose activation can initiate molecular events in the carcinogenesis process. These receptors include the aryl hydrocarbon receptor (AhR), peroxisome proliferator-activated receptors (PPARs), estrogen and/or androgen receptors (ERs; AR), thyroid hormone receptors (THRs), and glucocorticoid receptors (GR).

## 2 Electrophiles or metabolically activated toxins that induce stemness

The KCC concept identifies electrophiles and metabolically activated toxins. Electrophiles, characterized by their electron deficiency, react with nucleophiles through covalent bonding and form adducts with vital cellular macromolecules, such as DNA. This interaction is central to carcinogenesis. While some carcinogens act directly as electrophiles, others undergo transformation into reactive metabolites by enzymes, such as cytochrome P450s, becoming potent carcinogens (15, 16).

PAHs and NPAHs require metabolic activation to generate electrophilic products that can form DNA adducts. AhR orchestrates the bioactivation and detoxification of activated metabolites. Beyond a certain threshold, detoxification capacity is overwhelmed and adaptive responses become maladaptive, implicating disrupted immune and metabolic pathways in genetic instability (17, 18). Therefore, electrophilicity not only contributes to genomic instability, but also influences the immune response and metabolism.

Both PFASs and some phthalate metabolites are considered electrophilic (19, 20) and have the potential to covalently bind to cellular macromolecules. The electrophilic nature of PFAS has been a topic of debate. While some experts argue that PFAS are not classically electrophilic due to their strong carbon-fluorine bonds and stability ( (19), accumulating evidence suggests that PFAS can undergo oxidation/reduction reactions, leading to the formation of reactive intermediates. These intermediates may interact with nucleophilic sites in biological molecules, emphasizing the potential for oxidative stress and its implications in carcinogenesis (21–23). PFAS compounds exhibit different binding behaviors depending on their carbon chain lengths and functional groups. For instance, new classes of PFAS that feature shorter chains and incorporate oxygen molecules are considered to be more reactive.

OPFRs are electrophilic compounds that react with nucleophiles in biological systems (24). As a compound containing bromine atoms, TDBPP can act as an electrophile by seeking electron-rich species to form covalent bonds.

Arsenic is commonly found in the environment as oxides, existing in trivalent or pentavalent forms, with a high affinity for electron-rich groups in biological molecules, such as thiols in detoxification pathways. This disrupts cellular processes and contributes to carcinogenesis and genotoxicity (25).

## 3 Can cause genomic instability

Genotoxic substances that inflict DNA damage do not always directly result in mutations, raising the question about their classification as carcinogens. Such damage can take various forms, including DNA adducts, strand breaks, or base modifications, which differ fundamentally from mutations that alter the DNA sequence itself, often as a byproduct of repair processes. This distinction underscores the importance of considering genotoxicity alongside the capacity to disrupt DNA repair mechanisms or induce genomic instability as a critical characteristic of carcinogens, aligning with the cancer hallmark of genomic instability. The intersection of genotoxic and mutagenic properties of carcinogens recognized by the IARC (12) and NTP (13) suggests the relevance of these agents in precipitating cancer, especially when considering individuals with a predisposition to genomic instability, such as those with hereditary syndromes that heighten vulnerability to additional environmental insults (26). This aligns with Knudson’s two-hit hypothesis (26)., which posits that the path to malignancy often requires multiple genetic insults, highlighting the complexity of cancer development and potential role of environmental toxins in precipitating germline mutations.

The linkage between hereditary cancer syndromes and genomic instability, whether chromosomal (CIN) or microsatellite (non-CIN), through mutations in DNA repair genes exemplifies the intricate relationship between genetic predisposition and cancer risk. (27). For instance, mutations in mismatch repair genes in Lynch syndrome or biallelic mutations in the MYH gene are associated with base excision repair pathways, leading to an elevated rate of G•C to T•A transversions and underlining the critical impact of DNA repair fidelity on cancer susceptibility (28)

Moreover, the role of oxidative stress induced by various carcinogens in contributing to genomic instability further emphasizes the need for a nuanced understanding of carcinogenesis. Oxidative stress can precipitate DNA damage, leading to genomic instability and the accumulation of mutations that facilitate cancer progression by enabling cells to acquire additional malignant traits. Thus, the identification of genotoxic agents and those inducing oxidative stress in genes crucial for DNA damage recognition, repair initiation, or damage prevention, is essential in the context of carcinogenesis. DNA double-strand breaks (DSBs) or interstrand crosslinks have been identified as contributing to an increased susceptibility to a spectrum of cancers, including, but not limited to, breast and ovarian cancer, leukemia, and lymphomas (29–31) Moreover, mutations in genes associated with nucleotide excision repair pathways have been implicated in predisposing individuals to skin cancer (32). There is an acute need to screen for agents specifically causing genomic instability in gatekeeper genes, which play a central role in maintaining genomic integrity.

In addition to their direct interactions with DNA and mutagenic effects, PAHs can induce genetic instability by producing reactive oxygen and nitrogen species (ROS/RNS), which can damage cellular components such as lipids, proteins, and DNA. PAHs also disrupt cell antioxidant defense mechanisms, including the depletion of antioxidant molecules such as glutathione and the inhibition of enzymes such as superoxide dismutase and catalase (33).

PFASs, phthalate metabolites, such as mono(2-ethylhexyl) phthalate (MEHP), and OPFRs can induce oxidative stress through the direct generation of ROS/RNS, or by inhibiting mitochondrial function, leading to an imbalance between ROS production and antioxidant defense mechanisms (34–36). Additionally, they can deplete cellular antioxidants such as glutathione and disrupt antioxidant enzyme activity (36).

Arsenic, particularly in its interconverted forms arsenite (As^III) and arsenate (As^V), undergoes redox reactions within cells, leading to the generation of ROS and subsequent oxidative stress. Arsenite, in particular has been identified as a potent inducer of oxidative stress through mechanisms such as mitochondrial dysfunction and the inhibition of antioxidant enzymes. These effects result in oxidative damage to cellular components, contributing to cellular dysfunction and toxicity (37).

Therefore, all these chemicals considered can induce genetic instability by generating oxidative stress, thereby fostering inflammation (38).

## 4 Induces epigenetic alterations

The rapid assessment of epigenetic effects is essential for both short-term and long-term consequences of toxin exposure, considering that the KCCs and hallmarks both propose epigenetic dysregulation and non-mutational epigenetic reprogramming (39). Studies on non-smoking Polish coke-oven workers exposed to PAHs found alterations in DNA methylation, including increased global and IL-6 gene methylation, and reduced methylation of p53 and HIC1 tumor suppressor genes. p53 hypomethylation is linked to chromosomal instability and higher micronuclei levels, suggesting that DNA methylation modifications are potential biomarkers of cancer risk due to PAH exposure (40).

PFASs can induce epigenetic alterations associated with childhood cancers, such as ependymomas (19, 41, 42). When combined with a high-fat diet, PFASs can support prostate cancer progression through epigenetic, transcriptomic, and metabolomic alterations, indicating a complex interplay between metabolism and epigenetics during cancer development (43, 44).

Phthalate exposure can alter DNA methylation and miRNA production and induce transgenerational epigenetic changes that affect transgenerational disease susceptibility (45, 46).

OPFRs exposure is associated with alterations in DNA methylation patterns and histone modifications (47).

Arsenic metabolism involves methylation reactions that share similarities with DNA methylation pathways, suggesting a potential interplay between arsenic metabolism and DNA methylation. Arsenic exposure can cause global changes in DNA methylation, and is associated with prostate cancer (48, 49).

Epigenetic changes can affect receptors and trigger molecular events that may cause cancer. DNA methylation patterns can silence or alter receptor gene expression, thereby affecting normal signaling. Histone modifications can change the chromatin structure and influence receptor expression by impacting promoter accessibility. mRNAs can regulate receptor expression by targeting messenger RNAs to degrade or inhibit their translation. Although some EDCs may disrupt epigenetic programming during development, it remains unclear whether this leads to negative outcomes (50) This emphasizes the pivotal role of receptor-mediated effects in the context of KCCs, highlighting how epigenetic changes contribute to receptor dysfunction and subsequent carcinogenic processes.

## 5 Induces chronic inflammation

Chronic inflammation, a key characteristic of carcinogenesis, is intricately linked to the hallmarks of tumor-promoting inflammation and alterations in the microbiome or polymorphic microbiomes. This association is due to both the direct effects of environmental toxins and indirect effects via changes in the microbiome at the population level.

### 5.1 Direct impact on immune-inflammatory responses

Research integrating single- and multiple-exposure models has shed light on the immunoinflammatory response to mixed chemical exposure, revealing the differential effects of chemicals on immune-inflammatory markers.

All chemicals that induce oxidative stress are potentially involved in the inflammatory processes.

The immune-inflammatory response to environmental exposure is mediated by AhR activation, leading to inflammasomes and adaptive responses. Chronic inflammation occurs when adaptive responses become maladaptive due to sustained or high exposure (17, 18).

PAHs and metals have been identified as significant influencers, underscoring the complexity of the health effects of multiple chemical exposures (41, 51) necessitating the development of sophisticated models to decipher the interactions and non-linear relationships between chemical co-exposure and immune-inflammatory responses.

### 5.2 Indirect impact through the microbiome

The ubiquitous Helicobacter pylori, co-evolved with humans for 50,000 years, represents the dual role of microbiota in health and disease. H. pylori is associated with a reduced risk of certain diseases and interacts with gut microbiota to influence metabolic processes (52, 53). However, its presence has also been implicated in a significant proportion of gastric cancers (54).

The microbiome impact on cancer progression supports Paget’s seed and soil hypothesis. The microbiome can alter the tumor microenvironment, thereby facilitating or hindering cancer development. This corresponds to the discovery that oncoviruses, such as the Rous sarcoma virus, necessitate a suitable “soil” for the oncogenic “seeds” to thrive (55–57).

Chronic inflammation, tumor-promoting inflammation, and altered microbiomes are key factors to consider when determining the environmental toxins that drive precursor lesions to malignancy. These factors exploit the extended latency period, as seen in colorectal cancer development (58), to prevent such progression.

Environmental pollutants, such as heavy metals, pesticides, and food additives, can harm gut microbiomes and potentially cause or exacerbate human diseases. This damage can result from both direct and indirect effects on gut bacteria, leading to alterations in the microbial diversity and metabolic processes.

One mechanism of microbiome toxicity is the changes in the microbial metabolites, which bind AhR or t (59) he Farnesoid X Receptor (FXR) (60), affecting the immune response and metabolism.

PFAS can lead to alterations in gut microbiota and reduce microbiome diversity (61, 62).

DEHP modifies mouse intestinal microbiota, affecting metabolism and intestinal integrity (63).

Arsenic affects microbiome composition and function, with microbial redox transformations influencing its fate and toxicity when inhaled or ingested (64–66).

## 6 Is immunosuppressive

Cancer immune evasion and immunosuppression have distinct, yet interrelated mechanisms and implications.

Immune evasion, a cancer hallmark, refers to tumor’s ability to avoid immune detection through various strategies, including immunosuppression. In metastatic melanoma treatments, such as adoptive cell transfer therapies, initial remission can be followed by relapse due to melanoma cell dedifferentiation influenced by proinflammatory cytokines such as TNF-α within the tumor microenvironment, facilitating immune evasion through antigen loss (67).

The distinction between immunosuppressive effects, such as those observed in organ transplantation, and immune evasion strategies, including camouflage of the immune system, prompts a revaluation of this key characteristic. Environmental toxins, while traditionally associated with immunosuppression, may also play roles in facilitating immune evasion, underscoring the need for a broader focus on environmental factors that hinder the body’s immunosurveillance mechanisms against cancers.

The role of AhR in both cancer immune surveillance and immune evasion makes it a potential target for disruption and tumor promotion by exogenous chemicals, such as PAHs (68).

PPARs regulate immune responses by controlling inflammation and immune cell activity. AhR, PPARs, and other nuclear receptors interact to enhance immunosuppression.

PFAS have emerged as a significant concern because of their potential to induce immunosuppression (69).

Other environmental contaminants, ranging from fungicides and herbicides to personal care substances and industrial agents such as DEHP, which affect cytokine secretion (70), have been implicated in potentially compromising tumor immunosurveillance.

## 7 Modulates receptor-mediated effects

The nexus between receptor-mediated signaling and perpetuation of cell proliferation underscores a fundamental aspect of carcinogenesis, emphasizing the critical role of receptor pathways in the broader context of cancer cell growth and beyond molecular initiating events. Many chemicals, such as PAHs, PFAS, and phthalates, interact with multiple receptors, leading to complex downstream effects. Recent reviews provide a broader spectrum of receptor-mediated pathways involved in these interactions (71–73).

An integrated approach to understand how the modulation of receptor-mediated pathways directly contributes to the characteristics of sustained proliferative signaling is crucial.

PFASs underscore the connection between receptor modulation and proliferative signaling because of their ability to act as PPARs agonists or antagonists.

Tetrabromobisphenol A (TBBPA), a widely used HFR, interacts with both ER and AR, leading to a combined effect (74), which has been proposed as a mechanism in the carcinogenesis of triple-negative breast cancer (TNBC) (75), and with THR (76, 77), modulating genes involved in thyroid cancer, through epigenetic alterations (77, 78).

Arsenite can impede GR-mediated transcription at non-toxic levels, impacting nuclear function without affecting hormone-induced activation or translocation (79) Other chemicals, such as benzophenone-1 (BP1), affect ER pathways, which regulate cell proliferation and cell cycle. BP1 stimulates ER-positive cancer cells and modulates cyclin D1 expression, highlighting the importance of these pathways in maintaining proliferative signaling (80).

Taken together, these insights argue for a paradigm that acknowledges that screening for environmental toxins that mediate receptor-mediated modulation must essentially focus on sustained proliferative signaling as one of the majors read out (81).

## 8 Causes immortalization: altered lengthening of telomeres & evasion of cell death

The focus on immortalization and evasion of apoptosis, two critical hallmarks, contributes significantly to the understanding of environmental toxin-driven cancer via KCCs.

Telomere dynamics, influenced by environmental factors such as bisphenol A (TBBPA) and persistent organic pollutants (POPs), underscores the role of toxins in aging and disease susceptibility (82, 83). Investigations of telomere alterations among astronauts and arsenic exposure further highlight the complex interplay between toxins and telomere length regulation (84, 85).

Disrupted regulation of apoptosis by environmental mutagens, including endocrine-disrupting chemicals (EDCs) such as bisphenol A, is implicated in cancer development (86). Climate change exacerbates this risk by altering environmental stressors, contributing to air pollution complexity, and disrupting the apoptotic signaling pathway (87).

PAHs have been implicated in oral squamous cell carcinoma by influencing cell fate decisions and promoting cell immortalization during senescence (88).

Exposure to PFAS has been associated with altered plasma membrane fluidity, affecting calcium signaling and increasing platelet response to agonists, potentially influencing cell survival and evasion of cell death (89).

High arsenite concentrations may decrease telomerase activity and telomere length, leading to apoptosis (90).

## 9 Deregulating cellular energetics: a fulcrum for KCC - alters cell proliferation, cell death, or nutrient supply

The final key characteristic of carcinogens, instead of focusing on three different aspects, which have already been addressed via other characteristics, may benefit from specifically focusing on deregulating cellular energetics. This focus will be crucial for mitigating toxin exposure and cancer metabolic underpinnings.

PAHs offer a good example of how chemical exposure can deregulate cellular energetics, highlighting the intricate relationship between environmental toxins and cancer metabolism. PAHs can induce metabolic reprogramming by generating ROS and causing mitochondrial dysfunction. This leads to a shift towards glycolysis and away from oxidative phosphorylation, creating an environment favorable to cancer cell growth and survival (91). PAHs reactive intermediates such as diol epoxides can bind proteins leading to the generation of advanced glycation end products (AGEs) through the Maillard reaction (92). AGEs, interacting with their receptors (RAGE), induce metabolic disruption and histone glycation and trigger. the activation of key inflammatory signaling pathways (93). This mechanism has been confirmed in occupational exposure to PAHs, and metal fumes in shipyard welders (94).

PFAS are implicated in inducing epigenetic alterations and influencing cell proliferation, potentially contributing to cancer development (19).

Phthalates are associated with the redox control of cancer cell destruction, where factors such as insufficient oxygen and nutrients can lead to cell death in tumor masses (95).

OPFRs affect diverse molecular pathways controlling cell proliferation and death, potentially contributing to cancer development (96).

Arsenic exposure has been linked to alterations in the gut microbiome, which can influence nutrient supply and potentially contribute to cancer development (97).

## 10 Discussion

KCCs are intrinsic properties of chemical molecules that contribute to carcinogenesis initiation and sustenance. However, assessing whether these characteristics lead to adverse carcinogenic effects depends on factors such as the exposure concentration, immune system integrity, and tissue-specific response variability (17, 98, 99)(Chemical molecules may exhibit multiple characteristics depending on factors such as the exposure route and the target organ. This complexity underscores the need to consider the interrelationships among KCCs and between KCCs and cancer hallmarks.

We examined chemicals previously evaluated for their potential to cause cancer and found that although the evidence of carcinogenicity varies in strength, they share similar KCCs. Our analysis r suggested that receptor activation is a primary molecular event driving the mechanisms supported by KCCs. Receptor binding plays a crucial role in activating other KCCs, including electrophilicity, immune response disruption, oxidative stress, and inflammation, highlighting the connections between KCCs and their role in cancer initiation.

**Figure 1:**
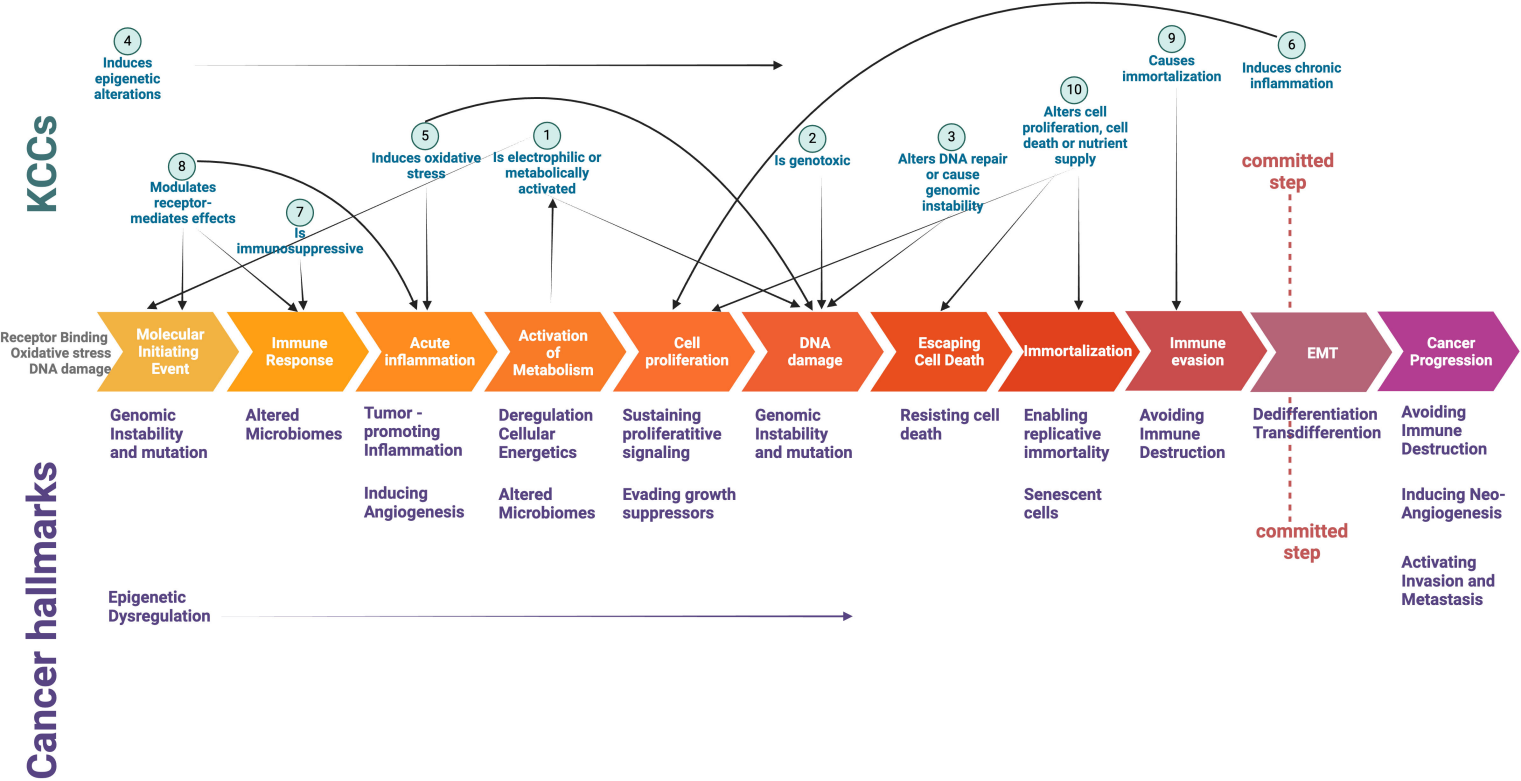
Intersection of Key Characteristics of Carcinogens (KCCs) and Cancer Hallmarks in a schematic depiction of critical events underlying cancer initiation and progression. The committed step indicates the critical transition towards a full malignant phenotype (see ref #^73^). *Created with Biorender.com*.

This complexity highlights the need to study the links between KCCs and cancer hallmarks, investigating how chemical molecule behavior aligns with hallmark responses (e.g., pathway analysis for common downstream events, multi-omics and spatial data analysis combined with phenotypic activity). The OECD Integrated Approach for Testing and Assessment (IATA) for non-genotoxic carcinogens integrates multiple sources of information and data to assess the carcinogenic potential of a substance and incorporates cancer hallmarks as part of the evaluation process of chemical substances (98, 99). It is rooted in the Adverse Outcome Pathway (AOP) framework, to use key events and molecular pathways in carcinogenesis to pinpoint chemical targets crucial for sustaining cancer progression (98, 99). From this perspective, KCCs appear to be associated with early events that manifest at the molecular or cellular level, but do not involve processes more directly linked to cellular transformation towards malignancy, such as dedifferentiation or transdifferentiation, and the plasticity stemming from cytoskeletal rearrangement to epithelial-mesenchymal transition (**Figure 1**).

## 11 Perspective

While KCCs may relate to a chemical’s potential to initiate the carcinogenesis process, they do not encompass the cellular context’s ability to adapt, show resilience, or mount defense mechanisms. Examining the transition from adaptive to potentially harmful responses could offer further clarity in assessing carcinogenic risks.

To ensure the accuracy of testing, it is important to incorporate the ability to identify malignancy (81, 100). For instance, microenvironment changes can help identify the early influences of carcinogens in promoting tumorigenesis (81, 101, 102) and serve as biological markers of chemical exposure (103). This ensures a comprehensive evaluation that not only identifies the initial stages of carcinogenesis, but also captures the ultimate endpoint of malignancy, providing a system toxicology-oriented and more holistic understanding of the carcinogenic potential of the tested chemicals. This aligns with the Carcinogenicity Health Effects Innovation Program’s goal (104), part of the new NIEHS FY 2025-2029 Strategic Plan, of creating a deeper understanding of the mechanisms through which environmental exposures affect biological processes leading to cancer disease (104).

The cancer hallmarks have greatly improved our knowledge of the mechanisms underlying cancer. However, toxicologists require a comprehensive framework that not only identifies carcinogenic agents using the KCCs, but also incorporates the intricate principles of cancer hallmarks. This integration is crucial for creating a robust methodology that can proactively detect potential carcinogens.

Targeted identification of prevalent carcinogenic toxins can be facilitated by integrating the KCC concept with hallmark-based mechanisms, thereby enabling the development of zone-specific prevention tactics. This methodology paves the way for precision toxicology utilizing modern technologies, including artificial intelligence, to screen segregated zones using an integrated framework composed of the KCCs and cancer hallmarks.

As our spatial-temporal comprehension of cancer deepens with the advent of sophisticated tools and methodologies, there is an opportunity to expand the existing and evolving hallmarks of cancer development and carcinogenesis. This enriches our conceptual model of disease and disease transition starting from pre-disease states. Toxicologists must integrate emerging hallmarks into a comprehensive set of key features, including the existing KCCs, to evaluate routine exposure to potential toxins and mitigate the global health impacts of cancer. Collaboration between KCCs and cancer hallmark communities and the development of a next-generation framework for methods such as NAMs and human exposure -based mechanistic biomarkers are vital for toxicology and cancer research. It is essential to advance cancer prevention strategies, precision environmental health, and align research with the regulatory requirements and global public health needs.

## Data Availability

The original contributions presented in the study are included in the article/**Supplementary Material**. Further inquiries can be directed to the corresponding author.

## References

[1] Hanahan D Weinberg RA. The hallmarks of cancer. Cell. (2000) 100:57–70. doi: 10.1016/S0092-8674(00)81683-9 10647931

[2] Hanahan D Weinberg RA. Hallmarks of cancer: the next generation. Cell. (2011) 144:646–74. doi: 10.1016/j.cell.2011.02.013 21376230

[3] Senga SS Grose RP. Hallmarks of cancer-the new testament. Open Biol. (2021) 11:200358. doi: 10.1098/rsob.200358 33465324 PMC7881179

[4] Hanahan D. Hallmarks of cancer: New dimensions. Cancer Discovery. (2022) 12:31–46. doi: 10.1158/2159-8290.CD-21-1059 35022204

[5] Wild CP. Complementing the genome with an "exposome": the outstanding challenge of environmental exposure measurement in molecular epidemiology. Cancer Epidemiol Biomarkers Prev. (2005) 14:1847–50. doi: 10.1158/1055-9965.EPI-05-0456 16103423

[6] Miller GW. The exposome at NIEHS: from workshops to manuscripts. Exposome. (2023) 3:osad011. doi: 10.1093/exposome/osad011 38045731 PMC10689254

[7] Goodson WH 3rd Lowe L Carpenter DO Gilbertson M Manaf Ali A Lopez de Cerain Salsamendi A. Assessing the carcinogenic potential of low-dose exposures to chemical mixtures in the environment: the challenge ahead. Carcinogenesis. (2015) 36 Suppl 1:S254–96. doi: 10.1093/carcin/bgv039 PMC4480130 26106142

[8] Smith MT Guyton KZ Gibbons CF Fritz JM Portier CJ Rusyn I. Key characteristics of carcinogens as a basis for organizing data on mechanisms of carcinogenesis. Environ Health Perspect. (2016) 124:713–21. doi: 10.1289/ehp.1509912 PMC4892922 26600562

[9] Smith MT Guyton KZ Kleinstreuer N Borrel A Cardenas A Chiu WA. The key characteristics of carcinogens: Relationship to the hallmarks of cancer, relevant biomarkers, and assays to measure them. Cancer Epidemiol Biomarkers Prev. (2020) 29:1887–903. doi: 10.1158/1055-9965.EPI-19-1346 PMC7483401 32152214

[10] IARC. IARC Preamble as Amended in 2019. Monographs I, editor. Lyon, France: International Agency for Research on Cancer (2021).

[11] Bisson W Arroyave W Atwood S Jahnke G Mehta S Schwingl P. Framework for evaluating the level of evidence of carcinogenicity from mechanistic studies: The Report on Carcinogens Handbook. The Toxicologist (Supplement to Toxicological Sciences (2024) (SO, Reston VA), pp 75–6. Available at: https://www.toxicology.org/pubs/docs/Tox/2023Tox.pdf (Accessed August 2024).

[12] IARC. Agents classified by the IARC Monographs Vol. 1–135. Monographs I, editor. Lyon, France: International Agency for Research on Cancer (2023).

[13] NTP. Report on Carcinogens. Fifteenth Edition. Research Triangle Park, NC, USA: National Toxicology Program Department of Health and Human Services, Public Health Service (2021).

[14] Zahm S Bonde JP Chiu WA Hoppin J Kanno J Abdallah M. Carcinogenicity of perfluorooctanoic acid and perfluorooctanesulfonic acid. Lancet Oncol. (2024) 25:16–7. doi: 10.1016/S1470-2045(23)00622-8 PMC12183505 38043561

[15] Batal M Boudry I Mouret S Clery-Barraud C Wartelle J Berard I. DNA damage in internal organs after cutaneous exposure to sulphur mustard. Toxicol Appl Pharmacol. (2014) 278:39–44. doi: 10.1016/j.taap.2014.04.003 24732442

[16] Smith MT. The mechanism of benzene-induced leukemia: a hypothesis and speculations on the causes of leukemia. Environ Health Perspect. (1996) 104 Suppl 6:1219–25 doi: 10.1289/ehp.961041219. PMC1469721 9118896

[17] Mascolo MG Perdichizzi S Vaccari M Rotondo F Zanzi C Grilli S. The Transformics Assay: first steps for the development of an integrated approach to investigate the Malignant cell transformation in vitro. Carcinogenesis. (2018) 39:955–67. doi: 10.1093/carcin/bgy037 PMC6031005 29554273

[18] Pillo G Mascolo MG Zanzi C Rotondo F Serra S Bortone F. Mechanistic interrogation of cell transformation *in vitro*: The transformics assay as an exemplar of oncotransformation. Int J Mol Sci. (2022) 23:7603. doi: 10.3390/ijms23147603 35886950 PMC9321586

[19] Temkin AM Hocevar BA Andrews DQ Naidenko OV Kamendulis LM. Application of the key characteristics of carcinogens to per and polyfluoroalkyl substances. Int J Environ Res Public Health. (2020) 17. doi: 10.3390/ijerph17051668 PMC7084585 32143379

[20] Goutte A Alliot F Budzinski H Simonnet-Laprade C Santos R Lachaux V. Trophic transfer of micropollutants and their metabolites in an urban riverine food web. Environ Sci Technol. (2020) 54:8043–50. doi: 10.1021/acs.est.0c01411 32496759

[21] Grandjean P Clapp R. Perfluorinated alkyl substances: emerging insights into health risks. New solutions: J Environ Occup Health policy. (2015) 25:147–63. doi: 10.1177/1048291115590506 PMC6172956 26084549

[22] Sunderland EM Hu XC Dassuncao C Tokranov AK Wagner CC Allen JG. A review of the pathways of human exposure to poly- and perfluoroalkyl substances (PFASs) and present understanding of health effects. J Expo Sci Environ Epidemiol. (2019) 29:131–47. doi: 10.1038/s41370-018-0094-1 PMC6380916 30470793

[23] Peng M Xu Y Wu Y Cai X Zhang W Zheng L. Binding affinity and mechanism of six PFAS with human serum albumin: Insights from multi-spectroscopy, DFT and molecular dynamics approaches. Toxics. (2024) 12. doi: 10.3390/toxics12010043 PMC10819430 38250999

[24] Yang J Zhao W Li Y. Human health risk regulation of reproductive toxicity, neurotoxicity, and endocrine disruption in special populations exposed to organophosphorus flame retardants. Expo Health. (2021) 13:551–66. doi: 10.1007/s12403-021-00402-y

[25] Danes JM Palma FR Bonini MG. Arsenic and other metals as phenotype driving electrophiles in carcinogenesis. Semin Cancer Biol. (2021) 76:287–91. doi: 10.1016/j.semcancer.2021.09.012 34563651

[26] Knudson AG Jr. Mutation and cancer: statistical study of retinoblastoma. Proc Natl Acad Sci U S A. (1971) 68:820–3. doi: 10.1073/pnas.68.4.820 PMC389051 5279523

[27] Leach FS Nicolaides NC Papadopoulos N Liu B Jen J Parsons R. Mutations of a mutS homolog in hereditary nonpolyposis colorectal cancer. Cell. (1993) 75:1215–25. doi: 10.1016/0092-8674(93)90330-S 8261515

[28] Al-Tassan N Chmiel NH Maynard J Fleming N Livingston AL Williams GT. Inherited variants of MYH associated with somatic G:C–>T:A mutations in colorectal tumors. Nat Genet. (2002) 30:227–32. doi: 10.1038/ng828 11818965

[29] Bachrati CZ Hickson ID. RecQ helicases: suppressors of tumorigenesis and premature aging. Biochem J. (2003) 374:577–606. doi: 10.1042/bj20030491 12803543 PMC1223634

[30] Kennedy RD D'Andrea AD. DNA repair pathways in clinical practice: lessons from pediatric cancer susceptibility syndromes. J Clin Oncol. (2006) 24:3799–808. doi: 10.1200/JCO.2005.05.4171 16896009

[31] Ripperger T Gadzicki D Meindl A Schlegelberger B. Breast cancer susceptibility: current knowledge and implications for genetic counselling. Eur J Hum Genet. (2009) 17:722–31. doi: 10.1038/ejhg.2008.212 PMC2947107 19092773

[32] Cleaver JE. Cancer in xeroderma pigmentosum and related disorders of DNA repair. Nat Rev Cancer. (2005) 5:564–73. doi: 10.1038/nrc1652 16069818

[33] Ranchoux B Meloche J Paulin R Boucherat O Provencher S Bonnet S. DNA damage and pulmonary hypertension. Int J Mol Sci. (2016) 17. doi: 10.3390/ijms17060990 PMC4926518 27338373

[34] Ng F Berk M Dean O Bush AI. Oxidative stress in psychiatric disorders: evidence base and therapeutic implications. Int J Neuropsychopharmacol. (2008) 11:851–76. doi: 10.1017/S1461145707008401 18205981

[35] Henriksen EJ Diamond-Stanic MK Marchionne EM. Oxidative stress and the etiology of insulin resistance and type 2 diabetes. Free Radic Biol Med. (2011) 51:993–9. doi: 10.1016/j.freeradbiomed.2010.12.005 PMC3071882 21163347

[36] Potter SJ Kumar DL DeFalco T. Origin and differentiation of androgen-producing cells in the gonads. Results Probl Cell Differ. (2016) 58:101–34. doi: 10.1007/978-3-319-31973-5_5 27300177

[37] Jomova K Jenisova Z Feszterova M Baros S Liska J Hudecova D. Arsenic: toxicity, oxidative stress and human disease. J Appl Toxicol. (2011) 31:95–107. doi: 10.1002/jat.1649 21321970

[38] Meng Y Xu X Xie G Zhang Y Chen S Qiu Y. Alkyl organophosphate flame retardants (OPFRs) induce lung inflammation and aggravate OVA-simulated asthmatic response via the NF-small ka, CyrillicB signaling pathway. Environ Int. (2022) 163:107209. doi: 10.1016/j.envint.2022.107209 35358787

[39] Alegria-Torres JA Barretta F Batres-Esquivel LE Carrizales-Yanez L Perez-Maldonado IN Baccarelli A. Epigenetic markers of exposure to polycyclic aromatic hydrocarbons in Mexican brickmakers: a pilot study. Chemosphere. (2013) 91:475–80. doi: 10.1016/j.chemosphere.2012.11.077 23305747

[40] Pavanello S Bollati V Pesatori AC Kapka L Bolognesi C Bertazzi PA. Global and gene-specific promoter methylation changes are related to anti-B[a]PDE-DNA adduct levels and influence micronuclei levels in polycyclic aromatic hydrocarbon-exposed individuals. Int J Cancer. (2009) 125:1692–7. doi: 10.1002/ijc.24492 19521983

[41] Liu Y Eliot MN Papandonatos GD Kelsey KT Fore R Langevin S. Gestational perfluoroalkyl substance exposure and DNA methylation at birth and 12 years of age: A longitudinal epigenome-wide association study. Environ Health Perspectives. (2022) 130:037005. doi: 10.1289/EHP10118 PMC8911098 35266797

[42] Kumar S Michealraj A Kim L Rich J Taylor M. ETMM-08 metabolic regulation of the epigenome drives lethal infantile ependymoma. Neuro-Oncology Adv. (2021) 3:i15–i6. doi: 10.1093/noajnl/vdab024.064 PMC10782558 32445698

[43] Imir OB Kaminsky AZ Zuo QY Liu YJ Singh R Spinella MJ. Per- and polyfluoroalkyl substance exposure combined with high-fat diet supports prostate cancer progression. Nutrients. (2021) 13. doi: 10.3390/nu13113902 PMC8623692 34836157

[44] Boyd RI Ahmad S Singh R Fazal Z Prins GS Madak Erdogan Z. Toward a mechanistic understanding of poly- and perfluoroalkylated substances and cancer. Cancers (Basel). (2022) 14. doi: 10.3390/cancers14122919 PMC9220899 35740585

[45] Grindler NM Vanderlinden L Karthikraj R Kannan K Teal S Polotsky AJ. Exposure to phthalate, an endocrine disrupting chemical, alters the first trimester placental methylome and transcriptome in women. Sci Rep. (2018) 8:6086. doi: 10.1038/s41598-018-24505-w 29666409 PMC5904105

[46] Thorson JLM Beck D Ben Maamar M Nilsson EE Skinner MK. Ancestral plastics exposure induces transgenerational disease-specific sperm epigenome-wide association biomarkers. Environ Epigenet. (2021) 7:dvaa023. doi: 10.1093/eep/dvaa023 33841921 PMC8022921

[47] Maury E Hashizume R. Epigenetic modification in chromatin machinery and its deregulation in pediatric brain tumors: Insight into epigenetic therapies. Epigenetics. (2017) 12:353–69. doi: 10.1080/15592294.2016.1278095 PMC5453195 28059591

[48] Reichard JF Schnekenburger M Puga A. Long term low-dose arsenic exposure induces loss of DNA methylation. Biochem Biophys Res Commun. (2007) 352:188–92. doi: 10.1016/j.bbrc.2006.11.001 PMC1866367 17107663

[49] Treas JN Tyagi T Singh KP. Effects of chronic exposure to arsenic and estrogen on epigenetic regulatory genes expression and epigenetic code in human prostate epithelial cells. PLoS One. (2012) 7:e43880. doi: 10.1371/journal.pone.0043880 22952798 PMC3428278

[50] Jacobs MN Marczylo EL Guerrero-Bosagna C Rüegg J. Marked for life: epigenetic effects of endocrine disrupting chemicals. Annu Rev Environ Resources. (2017) 42:105–60. doi: 10.1146/annurev-environ-102016-061111

[51] Arroyave W Sethi M Lemeris C Hodgson ME Mehta S Lunn R. Mapping the mechanistic evidence of wood smoke and wildfire studies in humans. ISEE Conf Abstracts. (2022) 2022. doi: 10.1289/isee.2022.O-OP-203

[52] Amieva M Peek RM Jr. Pathobiology of helicobacter pylori-induced gastric cancer. Gastroenterology. (2016) 150:64–78. doi: 10.1053/j.gastro.2015.09.004 26385073 PMC4691563

[53] Blaser MJ Atherton JC. Helicobacter pylori persistence: biology and disease. J Clin Invest. (2004) 113:321–33. doi: 10.1172/JCI20925 PMC324548 14755326

[54] Usui G Matsusaka K Huang KK Zhu F Shinozaki T Fukuyo M. Integrated environmental, lifestyle, and epigenetic risk prediction of primary gastric neoplasia using the longitudinally monitored cohorts. EBioMedicine. (2023) 98:104844. doi: 10.1016/j.ebiom.2023.104844 38251469 PMC10755115

[55] Paget G. Remarks on a case of alternate partial anaesthesia. Br Med J. (1889) 1:1–3. doi: 10.1136/bmj.1.1462.1 PMC2154531 20752533

[56] Rous P. A sarcoma of the fowl transmissible by an agent separable from the tumor cells. J Exp Med. (1911) 13:397–411. doi: 10.1084/jem.13.4.397 19867421 PMC2124874

[57] Dolberg DS Bissell MJ. Inability of Rous sarcoma virus to cause sarcomas in the avian embryo. Nature. (1984) 309:552–6. doi: 10.1038/309552a0 6203040

[58] Fearon ER Vogelstein B. A genetic model for colorectal tumorigenesis. Cell. (1990) 61:759–67. doi: 10.1016/0092-8674(90)90186-I 2188735

[59] Tu P Chi L Bodnar W Zhang Z Gao B Bian X. Gut microbiome toxicity: Connecting the environment and gut microbiome-associated diseases. Toxics. (2020) 8:19. doi: 10.3390/toxics8010019 32178396 PMC7151736

[60] Sun L Xie C Wang G Wu Y Wu Q Wang X. Gut microbiota and intestinal FXR mediate the clinical benefits of metformin. Nat Med. (2018) 24:1919–29. doi: 10.1038/s41591-018-0222-4 PMC6479226 30397356

[61] Salihovic S Dickens AM Schoultz I Fart F Sinisalu L Lindeman T. Simultaneous determination of perfluoroalkyl substances and bile acids in human serum using ultra-high-performance liquid chromatography-tandem mass spectrometry. Anal Bioanal Chem. (2020) 412:2251–9. doi: 10.1007/s00216-019-02263-6 PMC7118038 31760452

[62] Laue HE Moroishi Y Palys TJ Christensen BC Criswell RL Peterson LA. Early-life exposure to per- and polyfluoroalkyl substances and infant gut microbial composition. Environ Epidemiol. (2023) 7:e238. doi: 10.1097/EE9.0000000000000238 36777525 PMC9916123

[63] Lei M Menon R Manteiga S Alden N Hunt C Alaniz RC. Environmental chemical diethylhexyl phthalate alters intestinal microbiota community structure and metabolite profile in mice. mSystems. (2019) 4. doi: 10.1128/mSystems.00724-19 PMC6906742 31822602

[64] Lu K Abo RP Schlieper KA Graffam ME Levine S Wishnok JS. Arsenic exposure perturbs the gut microbiome and its metabolic profile in mice: an integrated metagenomics and metabolomics analysis. Environ Health Perspect. (2014) 122:284–91. doi: 10.1289/ehp.1307429 PMC3948040 24413286

[65] Lu K Mahbub R Cable PH Ru H Parry NM Bodnar WM. Gut microbiome phenotypes driven by host genetics affect arsenic metabolism. Chem Res Toxicol. (2014) 27:172–4. doi: 10.1021/tx400454z PMC3997221 24490651

[66] McDermott TR Stolz JF Oremland RS. Arsenic and the gastrointestinal tract microbiome. Environ Microbiol Rep. (2020) 12:136–59. doi: 10.1111/1758-2229.12814 31773890

[67] Landsberg J Kohlmeyer J Renn M Bald T Rogava M Cron M. Melanomas resist T-cell therapy through inflammation-induced reversible dedifferentiation. Nature. (2012) 490:412–6. doi: 10.1038/nature11538 23051752

[68] Hill W Lim EL Weeden CE Lee C Augustine M Chen K. Lung adenocarcinoma promotion by air pollutants. Nature. (2023) 616:159–67. doi: 10.1038/s41586-023-05874-3 PMC7614604 37020004

[69] Fenton SE Ducatman A Boobis A DeWitt JC Lau C Ng C. Per- and polyfluoroalkyl substance toxicity and human health review: Current state of knowledge and strategies for informing future research. Environ Toxicol Chem. (2021) 40:606–30. doi: 10.1002/etc.4890 PMC7906952 33017053

[70] Hansen JF Nielsen CH Brorson MM Frederiksen H Hartoft-Nielsen ML Rasmussen AK. Influence of phthalates on in *vitro* innate and adaptive immune responses. PLoS One. (2015) 10:e0131168. doi: 10.1371/journal.pone.0131168 26110840 PMC4482536

[71] Bock KW. Aryl hydrocarbon receptor (AHR): From selected human target genes and crosstalk with transcription factors to multiple AHR functions. Biochem Pharmacol. (2019) 168:65–70. doi: 10.1016/j.bcp.2019.06.015 31228464

[72] Cheng HS Yip YS Lim EKY Wahli W Tan NS. PPARs and tumor microenvironment: The emerging roles of the metabolic master regulators in tumor stromal-epithelial crosstalk and carcinogenesis. Cancers (Basel). (2021) 13. doi: 10.3390/cancers13092153 PMC8125182 33946986

[73] Lee C. Collaborative power of nrf2 and PPARγ Activators against metabolic and drug-induced oxidative injury. Oxid Med Cell Longevity. (2017) 2017:1378175. doi: 10.1155/2017/1378175 PMC5591982 28928902

[74] Vera-Badillo FE Templeton AJ de Gouveia P Diaz-Padilla I Bedard PL Al-Mubarak M. Androgen receptor expression and outcomes in early breast cancer: A systematic review and meta-analysis. JNCI: J Natl Cancer Institute. (2013) 106. doi: 10.1093/jnci/djt319 24273215

[75] Honma N Matsuda Y Mikami T. Carcinogenesis of triple-negative breast cancer and sex steroid hormones. Cancers (Basel). (2021) 13. doi: 10.3390/cancers13112588 PMC8197527 34070471

[76] Ren X-M Yao L Xue Q Shi J Zhang Q Wang P. Binding and activity of tetrabromobisphenol A mono-ether structural analogs to thyroid hormone transport proteins and receptors. Environ Health Perspectives. (2020) 128:107008. doi: 10.1289/EHP6498 PMC7584160 33095664

[77] Otsuka S Ishihara A Yamauchi K. Ioxynil and tetrabromobisphenol a suppress thyroid-hormone-induced activation of transcriptional elongation mediated by histone modifications and RNA polymerase II phosphorylation. Toxicological Sci. (2014) 138(2):290–9. doi: 10.1093/toxsci/kfu012 24449421

[78] Liu M Li A Meng L Zhang G Guan X Zhu J. Exposure to novel brominated flame retardants and organophosphate esters and associations with thyroid cancer risk: A case–control study in eastern China. Environ Sci Technology. (2022) 56:17825–35. doi: 10.1021/acs.est.2c04759 36468700

[79] Kaltreider RC Davis AM Lariviere JP Hamilton JW. Arsenic alters the function of the glucocorticoid receptor as a transcription factor. Environ Health Perspect. (2001) 109:245–51. doi: 10.1289/ehp.01109245 PMC1240242 11333185

[80] Park MA Hwang KA Lee HR Yi BR Jeung EB Choi KC. Benzophenone-1 stimulated the growth of BG-1 ovarian cancer cells by cell cycle regulation via an estrogen receptor alpha-mediated signaling pathway in cellular and xenograft mouse models. Toxicology. (2013) 305:41–8. doi: 10.1016/j.tox.2012.12.021 23328252

[81] Colacci A Corvi R Ohmori K Paparella M Serra S Da Rocha Carrico I. The cell transformation assay: A historical assessment of current knowledge of applications in an integrated approach to testing and assessment for non-genotoxic carcinogens. Int J Mol Sci. (2023) 24. doi: 10.3390/ijms24065659 PMC10057754 36982734

[82] Liang J Shao Y Huang D Yang C Liu T Zeng X. Effects of prenatal exposure to bisphenols on newborn leucocyte telomere length: a prospective birth cohort study in China. Environ Sci pollut Res Int. (2023) 30:25013–23. doi: 10.1007/s11356-021-14496-z 34031828

[83] Roberts EK Boss J Mukherjee B Salerno S Zota A Needham BL. Persistent organic pollutant exposure contributes to Black/White differences in leukocyte telomere length in the National Health and Nutrition Examination Survey. Sci Rep. (2022) 12:19960. doi: 10.1038/s41598-022-24316-0 36402910 PMC9675834

[84] Luxton JJ McKenna MJ Taylor LE George KA Zwart SR Crucian BE. Temporal telomere and DNA damage responses in the space radiation environment. Cell Rep. (2020) 33:108435. doi: 10.1016/j.celrep.2020.108435 33242411

[85] Ferrario D Collotta A Carfi M Bowe G Vahter M Hartung T. Arsenic induces telomerase expression and maintains telomere length in human cord blood cells. Toxicology. (2009) 260:132–41. doi: 10.1016/j.tox.2009.03.019 19464579

[86] Egle A Harris AW Bath ML O'Reilly L Cory S. VavP-Bcl2 transgenic mice develop follicular lymphoma preceded by germinal center hyperplasia. Blood. (2004) 103:2276–83. doi: 10.1182/blood-2003-07-2469 14630790

[87] LaKind JS Overpeck J Breysse PN Backer L Richardson SD Sobus J. Exposure science in an age of rapidly changing climate: challenges and opportunities. J Expo Sci Environ Epidemiol. (2016) 26:529–38. doi: 10.1038/jes.2016.35 PMC5071542 27485992

[88] Stanford EA Ramirez-Cardenas A Wang Z Novikov O Alamoud K Koutrakis P. Role for the aryl hydrocarbon receptor and diverse ligands in oral squamous cell carcinoma migration and tumorigenesis. Mol Cancer Res. (2016) 14:696–706. doi: 10.1158/1541-7786.MCR-16-0069 27130942 PMC4987205

[89] Meneguzzi A Fava C Castelli M Minuz P. Exposure to perfluoroalkyl chemicals and cardiovascular disease: Experimental and epidemiological evidence. Front Endocrinol. (2021) 12. doi: 10.3389/fendo.2021.706352 PMC8298860 34305819

[90] Kim Y-D Jang S-J Lim E-J Ha J-S Shivakumar SB Jeong G-J. Induction of telomere shortening and cellular apoptosis by sodium meta-arsenite in human cancer cell lines. Anim Cells Systems. (2017) 21:241–54. doi: 10.1080/19768354.2017.1342691 PMC6138346 30460075

[91] Monteverde T Muthalagu N Port J Murphy DJ. Evidence of cancer-promoting roles for AMPK and related kinases. FEBS J. (2015) 282:4658–71. doi: 10.1111/febs.13534 26426570

[92] Maillard LC. Action des acides amines sur les sucres; formation de melanoidines par voie méthodique" [Action of amino acids on sugars. Formation of melanoidins in a methodical way]. Comptes Rendus. (1912) 154:.66–8.

[93] Uribarri J del Castillo MD de la Maza MP Filip R Gugliucci A Luevano-Contreras C. Dietary advanced glycation end products and their role in health and disease. Adv Nutr. (2015) 6:461–73. doi: 10.3945/an.115.008433 PMC4496742 26178030

[94] Lai C-H Chou C-C Chuang H-C Lin G-J Pan C-H Chen W-L. Receptor for advanced glycation end products in relation to exposure to metal fumes and polycyclic aromatic hydrocarbon in shipyard welders. Ecotoxicology Environ Safety. (2020) 202:110920. doi: 10.1016/j.ecoenv.2020.110920 32800255

[95] Hegedűs C Kovács K Polgár Z Regdon Z Szabó É Robaszkiewicz A. Redox control of cancer cell destruction. Redox Biol. (2018) 16:59–74. doi: 10.1016/j.redox.2018.01.015 29477046 PMC5842284

[96] Trembley JH Wang G Unger G Slaton J Ahmed K. Protein kinase CK2 in health and disease. Cell Mol Life Sci. (2009) 66:1858–67. doi: 10.1007/s00018-009-9154-y PMC4385580 19387548

[97] Griggs JL Chi L Hanley NM Kohan M Herbin-Davis K Thomas DJ. Bioaccessibility of arsenic from contaminated soils and alteration of the gut microbiome in an *in vitro* gastrointestinal model. Environmental Pollution. (2022) 309:119753. doi: 10.1016/j.envpol.2022.119753 35835276 PMC9667710

[98] Jacobs MN Colacci A Corvi R Vaccari M Aguila MC Corvaro M. Chemical carcinogen safety testing: OECD expert group international consensus on the development of an integrated approach for the testing and assessment of chemical non-genotoxic carcinogens. Arch Toxicol. (2020) 94:2899–923. doi: 10.1007/s00204-020-02784-5 PMC7395040 32594184

[99] Jacobs MN Colacci A Louekari K Luijten M Hakkert BC Paparella M. International regulatory needs for development of an IATA for non-genotoxic carcinogenic chemical substances. ALTEX. (2016) 33:359–92. doi: 10.14573/altex 27120445

[100] Corton JC Mitchell CA Auerbach S Bushel P Ellinger-Ziegelbauer H Escobar PA. A collaborative initiative to establish genomic biomarkers for assessing tumorigenic potential to reduce reliance on conventional rodent carcinogenicity studies. Toxicological Sci. (2022) 188:4–16. doi: 10.1093/toxsci/kfac041 PMC9238304 35404422

[101] Bisson WH Amedei A Memeo L Forte S Felsher DW. Tumor-promoting/associated inflammation and the microenvironment: A state of the science and new horizons. In: Translational Toxicology and Therapeutics: Windows of Developmental Susceptibility in Reproduction and Cancer Eds. Waters M. Hughes C. (2018) (John Wiley & Sons).

[102] Casey SC Vaccari M Al-Mulla F Al-Temaimi R Amedei A Barcellos-Hoff MH. The effect of environmental chemicals on the tumor microenvironment. Carcinogenesis. (2015) 36 Suppl 1:S160–83. doi: 10.1093/carcin/bgv035 PMC4565612 26106136

[103] Aguilar D Colacino JA. Single cell approaches to understand environmental impacts on aggressive breast cancers. Curr Opin Toxicology. (2024), 100459. doi: 10.1016/j.cotox.2024.100459

[104] NIH, NIEHS. Carcinogenicity Health Effects Innovation North Carolina. Available online at: https://www.niehs.nih.gov/research/atniehs/dtt/strategic-plan/health/carcinogenicity. (Accessed June 2024).

