# Supplementary material for: Key characteristics of carcinogens meet hallmarks for prevention-cutting the Gordian knot
Source: Front Oncol. 2024 Sep 10;14:1420687. doi: 10.3389/fonc.2024.1420687 (PMC11491790; doi:10.3389/fonc.2024.1420687)
Supplement: Supplementary file 1 [file Table1.pdf]

Supplementary Material

Key Characteristics of Carcinogens meet Hallmarks for prevention-cutting the Gordian knot

Sasi S. Senga[1], William H. Bisson[2], Annamaria Colacci[3,4*]

[1] Nuffield Department of Medicine, University of Oxford, Oxford, United Kingdom
[2] Integrative Toxicology and Cancer Prevention, Durham, United States
[3]Agency for Prevention, Environment and Energy, Emilia-Romagna Region (Arpae),, Bologna, Italy
[4]Alma Mater Institute on Healthy Planet – University of Bologna, Bologna, Italy

1 Supplementary Tables

Table 1S. Key Characteristics of Selected Carcinogens: A Comparative Analysis

	Classification		Key Characteristics of Carcinogens									
	NTP[a]	IARC[b]	Is electrophilic or metabolically activated	Is genotoxic	Induce DNA repair alteration or genomic instability	Induce epigenetic alterations	Induce oxidative stress	Cause chronic inflammation	Is immuno-suppressive	Modulates receptor-mediated effects	Causes immortalization	Alters cell proliferation, cell death or nutrient supply
Arsenic	Known	1	Yes, in its environmental state as an oxide	Yes through oxidative stress	Yes through oxidative stress	Yes	Yes conversion to arsenite (As^III) and arsenate (As^V)	Yes through oxidative stress	Suggestive evidence	Yes through GR	Yes	Yes
OPFRs	Reasonably anticipated (TDBPP)	2A (TDBPP)	Yes	Yes through oxidative stress	Yes through oxidative stress		Yes	Yes through oxidative stress	Limited information	Yes through ER and AR	Yes	Yes
PAHs	Reasonably anticipated (15 PAHs)	1* (B(a)P)	Metabolically activated to electrophilic intermediates	Adducts to DNA	Yes	Yes	Yes	Yes,through AhR-mediated pathways	Yes, yhtough AhR.mediated pathways	Yes through AhR	Yes	Yes
PFAS	Not Listed	1 (PFOA) 2B (PFOS)	YES	Yes through oxidative stress	Yes through oxidative stress	Yes	Yes	Yes, indirect	Yes	Yes through PPARs	Yes	Yes

Phthalates	Reasonably anticipated (DEHP)	2B (DEHP)	Metabolically activated to electrophilic intermediates	Yes through oxidative stress	Yes through oxidative stress	Yes	Yes	Yes, indirect	Yes, through PPARs	Yes through PPARs	No information	Yes

[a]National Toxicology Program (NTP) 15th Report on Carcinogens (NTP, 2021 Ref 13)
International Agency for Research on Cancer (IARC) volumes 1-135 (IARC, 2023 Ref 12)
Abbreviations: B(a)P = benzo (a)pyrene; HFRs = halogenated flame retardants; OPFRs: organophosphate flame retardants; PAHs = polycyclic aromatic hydrocarbons; PFAS = perfluoroalkyl substances; PFOA = perfluorooctanoic acid; PFOS = perfluorooctane sulfonate; TBBPA= Tetrabromobisphenol A; TDBPP = tris(2,3-dibromopropyl) phosphate
